# A Systematic Review of Staff Perspectives on Safety on Psychiatric Wards

**DOI:** 10.1111/nhs.70270

**Published:** 2025-12-18

**Authors:** Oladapo Akinlotan, Maria Dumitriu

**Affiliations:** ^1^ School of Nursing, Faculty of Health, Medicine & Social Care Anglia Ruskin University Chelmsford UK; ^2^ School of Medicine, Faculty of Health, Medicine & Social Care Anglia Ruskin University Chelmsford UK

**Keywords:** patient safety, psychiatric wards, safety, systematic review, ward safety

## Abstract

Patients on psychiatric wards encounter harm while receiving care, which leads to millions of fatalities every year. Understanding staff's perspectives on patients' safety on psychiatric wards is crucial for managing safety issues and concerns. This study aims to provide a reliable summary of the current evidence on staff's perspectives regarding safety on psychiatric wards. Studies were identified through systematic searches of six electronic databases. The characteristics of eligible studies were limited to peer‐reviewed qualitative research published in the English language within the last 10 years, which explored staff's perspective on patient safety in psychiatric wards. Seventeen studies met all the eligibility criteria. Data synthesis was performed using a thematic analysis approach, and four major themes were identified: perception of safety, safety interventions, therapeutic environment, staff and patients' safety. Patient safety on psychiatric wards is multifaceted, necessitating a balance between protection and autonomy, effective environmental design, compassionate care, and staff well‐being. Safety interventions must consider both patients' needs and the emotional and physical demands on staff to create a therapeutic and secure environment.

## Summary

- This is the first systematic review that examines safety on psychiatric wards from the perspective of the healthcare professionals.
- Patient safety on psychiatric wards is a multifaceted challenge requiring targeted strategies to prevent self‐harm, suicide, violence, aggression, sexual abuse and medication misuse.
- Patient safety on psychiatric wards encompasses both physical protection and psychological security and requires a balance between protection and autonomy, effective environmental design, compassionate care, and staff well‐being.

## 1 Introduction

Providing safe care within psychiatric wards is crucial to promoting patients' recovery (Thibaut et al. 2019), however, patients on psychiatric wards encounter harm while receiving care, which leads to millions of fatalities every year (Berzins et al. 2020). On the other hand, providing safe care within psychiatric wards can be complicated due to patients' risks, needs, and behaviors (Thibaut et al. 2019). Patient safety on psychiatric wards is a complex and multifaceted concept that requires careful attention from staff to ensure safe care (Kanerva et al. 2012).

According to the World Health Organization (2023), patient safety refers to preventing avoidable harm to patients and creating healthcare systems that minimize risks, reduce errors, and mitigate harm when mistakes happen. Common safety issues on psychiatric wards include physical safety (violence and aggression), Psychological safety (self‐harm and suicide), sexual safety, and medication misuse (Jenkin et al. 2022; Berg et al. 2017). The use of physical restraint and seclusion on psychiatric wards have been associated with psychological and physical harm to the patients and unsafe restraint practice regularly results in injury and trauma to the patients (Jenkin et al. 2022).

Previous studies have highlighted that staff's perceptions of safety are influenced by the presence of workplace aggression and the physical design of wards (Haines et al. 2018; Mohammadi‐Gorji et al. 2020). Haines et al. (2018) found that most staff members felt encouraged to report aggressive incidents and this resulted in an improved perception of patient safety (Haines et al. 2018). On the other hand, studies that explored patients' perspectives described how restrictive environments and physical confinement led to unmet needs for care and autonomy, and triggered violence (Melvin et al. 2022).

Recent reviews have examined patients' perspectives on safety within psychiatric wards (Akinlotan et al. 2025; Weber et al. 2022). Akinlotan et al. (2025) found that patients perceive safety as a delicate balance involving the physical environment, staff behaviors, and ward practices. Similarly, Weber et al. (2022), highlighted the significance of factors such as crowding, privacy, and control over space in shaping patients' sense of safety. However, none of these reviews have explored staff's perspectives regarding patients' safety on psychiatric wards. This current systematic review aims to provide a reliable summary of the current evidence on staff's perspectives regarding patients' safety on psychiatric wards. This review is important because understanding staff's perspectives on patients' safety on psychiatric wards is crucial for managing safety issues and concerns, as this understanding will provide invaluable insights that inform practice and policy (Rhodes et al. 2016). To guide this review, the PEO framework (Hosseini et al. 2024) has been used (P‐Population: patients in psychiatric settings/wards; E‐Exposure: patient safety; O‐Outcome: staff perspectives).

## 2 Methodology

This systematic review was conducted in accordance with the Preferred Reporting Items for Systematic Reviews and Meta‐Analysis (PRISMA) guidelines (Page et al. 2021) (Appendix S1) and was registered with PROSPERO under the reference number CRD420251055484. The review was assessed for risk of bias via using Risk Of Bias In Systematic Reviews (ROBIS) tool (Whiting et al. 2016) (Appendix A). Meta‐analysis was not considered because all the included studies were qualitative in nature with no statistical components.

### 2.1 Search Strategy

Studies were identified through systematic searches of six electronic databases: MEDLINE, APA PsychInfo, CINAHL Plus with Full Text, Psychology and Behavioral Sciences Collection, APA PsycArticles, and Embase. The search was conducted in June 2025. Key search terms were derived from the title and used Boolean operator “OR” for similar terms, and Boolean operator “AND” for terms with different meaning to refine the search. The search terms used were: “patient* safety” OR “patient*” OR “safety” OR “adverse event*” AND “psychiatric” OR “mental health” AND “ward*” OR “hospital*” OR “acute setting*” OR “inpatient” AND “staff or nurse*” OR “healthcare professional*” OR “doctor*” OR “healthcare assistant*” OR “nursing assistant*” AND “perspective*” OR “view*” OR “perception*” OR “attitude*” OR “opinion” OR “understanding” OR “experience*.”

### 2.2 Eligibility Criteria

Eligible studies are limited to peer‐reviewed qualitative research published in the English language within the last 10 years and must be primary research studies. Eligible studies must focus on adult patients in any inpatient mental health setting, where the staff's perspective on patients' safety is explored separately from other populations. The staff needs to include mental health professionals of any age, gender, or work experience, and the research must have a significant focus on the safety of psychiatric inpatients. Further details about the eligibility criteria are provided in Table 1.

**TABLE 1:** Eligibility criteria for the selected studies.

	Inclusion criteria	Exclusion criteria
Study design	Primary research studies.	Secondary research studies, for example, systematic reviews.
Publication characteristics	Peer‐reviewed studies. Qualitative studies or mixed‐method studies with significant and distinct qualitative aspects. Published in the English language. Published within the last 10 years.	Non‐peer‐reviewed studies. Non‐qualitative studies. Published in a non‐English language. Published more than 10 years ago.
Population	Research focused on psychiatric patients who are adults. Research focused on patients in any inpatient mental health setting, for example, wards. Research focused on staff's perspectives. Mental health professionals of any age, gender, or work experience.	Research focused on psychiatric patients who are children or adolescents. Research focused on patients who are not in an inpatient mental health setting, for example, the community. Research focused on the perspectives of patients or caregivers, without that of the staff.
Exposure	Research with a major focus on the safety of psychiatric inpatients.	Research that did not have a major focus on patient safety on psychiatric wards. Research that did not present a discussion around patient safety, separately from other issues.
Outcome	Research that explored staff's perspectives, experiences, or perceptions of patient safety on psychiatric wards. Research that presented staff's perspectives on psychiatric wards separately from other populations, for example, patients or caregivers.	Research that did not explore staff's perspectives, experiences, or perceptions of patient safety on psychiatric wards. Research that did not present staff's perspectives on psychiatric wards separately from patients' or caregivers' perspectives.

### 2.3 Selection Process

**FIGURE 1:**
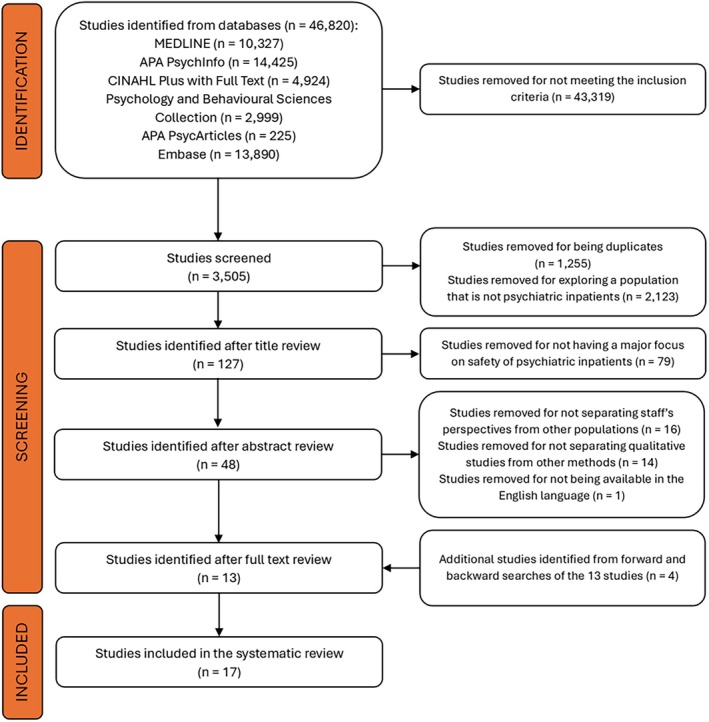
PRISMA flow diagram for the study.

The initial search identified 46 820 studies (Figure 1). From the initial search, 43 319 studies were excluded for not meeting the eligibility criteria, and 1255 duplicates were identified and removed. The remaining 3505 studies underwent careful screening of their titles and abstracts. As a result, 48 studies were selected for full‐text review. One study was removed because it is not available in the English language, 16 studies were excluded for failing to separate staff's perspectives from those of other populations, and 14 studies were excluded for not separating qualitative results from other findings. For the 13 remaining studies at this stage, forward and backward searches of the reference lists were conducted, and an additional four studies were selected that met all the inclusion criteria. A total of 17 studies were included in this review (Figure 1). Two independent reviewers completed the entire selection process. An agreement was reached at every screening stage to exclude studies. The two reviewers discussed any discrepancies in the screening process, and a consensus was reached. The two reviewers reached an agreement on the final selected studies.

### 2.4 Quality Assessment

The characteristics of all included studies are presented in Table 2. A quality assessment of all 17 selected studies was conducted using the Critical Appraisal Skills Programme (CASP 2018) (Table 3) and the Mixed Methods Appraisal Tool (MMAT) (Hong et al. 2018) (Table 4). The CASP 2018 tool revealed a generally high methodological rigor. Most studies received “Yes” responses across most CASP criteria, indicating clear statements of aims and findings, strong study designs, and appropriate methods of data collection and analysis. Notably, a concern emerged regarding the relationship between researchers and participants. Nine out of the 17 studies received a “No” response to the question of whether this relationship was adequately considered. MMAT assessment for qualitative studies resulted in all “Yes” responses across all studies, indicating a high level of methodological rigor and quality.

**TABLE 2:** Characteristics of all selected studies.

Author(s), Year, Country	Objectives	Participants (P), Setting (S), Age (A), Staff gender (SG), Work experience (WE)	Data collection (DC), Data analysis (DA)	Key findings	Limitations
Fallahi‐Khoshknab et al. (2018) Iran	To investigate nurses' opinions and experiences about patient safety in inpatient psychiatric wards.	P = 19 (supervisors, head nurses, and registered nurses) S: acute care wards in psychiatric hospitals A: not specified SG: female (9), male (10) WE: > 3 years	DC: in‐person interviews DA: qualitative content analysis	Patient safety depends on managing dangerous concealment, access control, adequate nurse staffing, proper knowledge and skills, and suitable facilities.	Some experiences that evoke unpleasant feelings were omitted, and certain events were forgotten over time.
Albutt et al. (2021) UK	To explore mental health professionals' perceptions of patient safety issues across community and inpatient mental health services.	P = 14 (nursing managers, registered mental health nurses, pharmacists, psychologists, occupational therapists, and social workers) S: inpatient mental health settings A: not specified SG: female (10), male (4) WE: 5 to 32 years	DC: phone interviews DA: framework analysis	Safety issues include organizational culture affecting incident reporting, communication barriers in care planning and information sharing, and limited access to treatment and services.	Small‐scale exploratory study with preliminary findings and participants were recruited via Twitter.
Alshowkan and Gamal (2019) Saudi Arabia	To explore psychiatric nurses' perceptions of patient safety at one hospital in Saudi Arabia.	P = 9 (nurses) S: psychiatric hospital A: 28 to 58 years SG: female (5), male (4) WE: 1 to 20 years	DC: in‐person interviews DA: thematic analysis	The head nurse is crucial for patient safety; staff‐patient safety is interconnected, and preventing harmful patient behavior is essential.	Participants were small sample of nurses limited to psychiatric wards in a tertiary hospital.
Barr et al. (2019) Australia	To determine how nurses' experiences and skill set can inform practice changes to reduce the use of restrictive practices.	P = 32 (nurses) S: state forensic mental health service A: 20 to ≥ 51 years SG: female (16), male (16) WE: < 2 to > 21 years	DC: in‐person interviews DA: qualitative content analysis	Higher aggression rates in forensic mental health inpatients lead to more restrictive nursing environments, requiring specialist skills like leadership, clinical supervision, and mentoring to ensure staff safety.	Conducted at a single forensic mental health service, limiting generalizability.
Berg et al. (2020a, 2020b) Norway	To explore safe clinical practice for suicidal patients hospitalized in mental health wards through understanding healthcare professionals' capacities to adapt to challenges and changes in clinical care.	P = 35 (nurses, doctors, psychologists) S: specialized mental health services in a university hospital A: not specified SG: female (28), male (7) WE: 1 to 24 years	DC: in‐person focus group and individual interviews DA: qualitative content analysis	Staff's adaptive capacities, like expertise, individualized care, and uncertainty management, are vital for safe clinical practice with patients hospitalized during suicidal crises.	There was a limited external validity due to single‐hospital setting and local organizational culture.
Hagen et al. (2017) Norway	To investigate mental health nurses' experiences of recognizing and responding to suicidal behavior/self‐harm and dealing with the emotional challenges in the care of potentially suicidal inpatients.	P = 8 (mental health nurses) S: psychiatric wards A: 43 to 60 years SG: female (7), male (1) WE: 5 to 25 years	DC: in‐person interviews DA: systematic text condensation	More experienced mental health nurses are more alert to suicidal cues due to their ability to recognize mental distress, and effective care requires balancing emotional involvement with professional distance.	First author's background as a mental health nurse may have influenced data collection and interpretation.
Hamaideh (2017) Saudi Arabia	To assess the perception of mental health nurses about patients' safety culture and to detect the factors that may affect patients' safety culture at psychiatric hospitals.	P = 224 (mental health nurses) S: psychiatric hospitals A: < 30 to ≥ 46 years SG: female (25), male (199) WE: ≥ 6 months	DC: questionnaires DA: qualitative content analysis	Mental health nurses rated patient safety as excellent or very good at 68.7%, acceptable at 20.5%, and poor or failing at 10.8%. Patient safety culture weakens with punitive error responses, poor communication, and inadequate staffing.	Sample included only mental health nurses and there may be potential bias from questionnaire distribution by managers and supervisors.
Hylén et al. (2018) Sweden	To describe the nursing staff and ward managers' experiences of safety and violence in everyday meetings with the patients.	P = 20 (17 nurses and assistant nurses, 3 managers) S: psychiatric inpatient care wards A: not specified SG: female, male WE: not specified	DC: in‐person focus groups and individual interviews DA: qualitative content analysis	The staff‐patient relationship is fundamental for effective care and for preventing and managing violence.	Only one ward per psychiatric specialty was included and focus groups may have inhibited participants from sharing private thoughts.
Kanerva et al. (2015) Finland	To explore the nursing staff's perceptions of patient safety in psychiatric inpatient care.	P = 26 (nurses) S: psychiatric hospital wards A: 23 to 69 years SG: female (16), male (10) WE: 1 to 30 years	DC: in‐person interviews DA: inductive content analysis	Patient safety is closely linked to medication care and maintaining nurses' professional skills. Nurses focused more on the skills needed for safe care than on suicide or seclusion and restraint practices.	Analyses performed by one researcher and some issues may not have been raised due to assumptions about the interviewer's prior knowledge.
Maddineshat et al. (2023) Iran	To explore a safe environment provided by mental health nurses in inpatient psychiatric wards in Iran.	P = 48 (mental health nurses) S: psychiatric wards A: median 37 years SG: female (22), male (26) WE: median 13 years	DC: in‐person interviews DA: qualitative content analysis	Vigilance is key to safety in Iran's psychiatric wards, but hyper‐vigilance leads to communication gaps and nurse burnout. Staff safety, security needs, and environmental hazards also challenge creating a safe environment.	Conducted in a single psychiatric hospital and small sample size may limit generalizability.
Marshall et al. (2019) Canada	To provide an in‐depth exploration of frontline forensic mental health staff's perceptions of safety on their units.	P = 88 (psychologists, psychiatrists, nurses, and social workers) S: minimum and medium security forensic inpatient units A: 22 to 63 years SG: female (65), male (23) WE: 3 months to 31 years	DC: in‐person interviews DA: thematic analysis	Open plan units, weighted furniture, and security officers enhance staff safety, while collaboration, support, and teamwork are essential for ensuring safety.	Voluntary participation may have led to a non‐representative staff sample and year‐long interviews could reflect varying hospital‐wide changes.
Olasoji et al. (2024) Australia	To explore the views of mental health nurses (MHNs) about the sexual safety of consumers receiving care in acute inpatient units.	P = 8 (mental health nurses) S: metropolitan acute inpatient unit A: not specified SG: not specified WE: not specified	DC: in‐person interviews DA: qualitative content analysis, followed by thematic analysis	Sexual safety in inpatient units is a shared responsibility, requiring patient awareness of expectations upon admission. Mental health units often lack proactive communication about patients' rights, and the environment can hinder maintaining sexual safety.	Conducted using single‐site hospital eight mental health nurses.
Sollied et al. (2023) Norway	To explore healthcare professionals' experiences with facilitating a safe and caring atmosphere in patients' everyday lives in forensic mental health wards.	P = 16 (nurses, occupational therapists, and social workers) S: forensic mental health care wards A: not specified SG: female (6), male (10) WE: > 4 years	DC: in‐person interviews DA: phenomenological hermeneutic analysis	Balancing daily activities to meet patients' needs fosters comfort and trust. Teamwork is essential for calmly resolving violence, alongside awareness of each patient's vulnerability and window of tolerance.	Datasets used are from 2013 and may not represent the most recent trends.
True et al. (2017) Unites States	To identify risk factors and protective factors in hospital‐based mental health settings that could improve the safety of persons with serious mental illness.	P = 20 (nurses, psychiatrists) S: inpatient psychiatric units in Veterans Health Administration acute care medical centers A: not specified SG: not specified WE: not specified	DC: in‐person interviews DA: qualitative content analysis	Threats to patient safety exist at system, provider, and patient levels. Protective factors include promoting a safety culture, advocating patient‐centeredness, and engaging leadership to support these changes.	Data was collected via phone interviews and organizational factors protecting patient safety were self‐reported by key informants.
Vahidi et al. (2018a, 2018b) Iran	To explore the link between therapeutic relationships and safety in Iranian psychiatric inpatient units.	P = 26 (19 nurses, 7 patients) S: psychiatric referral center A: 28 to 60 years SG: female (9), male (10) WE: 2 to 30 years	DC: in‐person interviews DA: qualitative content analysis	Supportive staff‐patient relationships empower patients to manage their thoughts, emotions, and behaviors, enhancing self‐efficacy, self‐control, and the safety of inpatient units.	Participants were predominantly nurses, under‐representing other healthcare professionals' perspectives.
Vahidi et al. (2018a, 2018b) Iran	To explore nurses' use of vigilance in the provision of inpatient psychiatric care.	P = 16 (nurses) S: psychiatric referral center A: 28 to 60 years SG: female (8), male (8) WE: 2 to 30 years	DC: in‐person interviews DA: qualitative content analysis	Vigilance is a subconscious, integral part of nurses' daily activities, recognizing the ward as an inherently risky environment.	Differences among nursing levels, such as experience, duration, were not explored.
Vogt et al. (2024) UK	To investigate how psychological safety is conceptualized by healthcare staff in inpatient mental health units, and what barriers and facilitators exist.	P = 12 (healthcare professionals with experience in adult mental health wards) S: adult inpatient mental health ward A: 24 to 31 years SG: female (12) WE: not specified	DC: online interviews DA: thematic analysis	Participants often felt physically unsafe at work, leading to psychological insecurity. Barriers included reliance on agency workers, punitive management, and risks of mental health work. Facilitators were proper staffing, meaningful relationships, and access to support.	All participants identified as female, potentially biasing emphasis on physical safety and not reflecting male experiences.

**TABLE 3:** Quality assessment of all selected studies based on Critical Appraisal Skills Programme (CASP 2018).

	1. Was there a clear statement of the aims of the research?	2. Is a qualitative methodology appropriate?	3. Was the research design appropriate to address the aims of the research?	4. Was the recruitment strategy appropriate to the aims of the research?	5. Was the data collected in a way that addressed the research issue?	6. Has the relationship between the researcher and participants been adequately considered?	7. Have ethical issues been taken into consideration?	8. Was the data analysis sufficiently rigorous?	9. Is there a clear statement of findings?	10. How valuable is the research?
Fallahi‐Khoshknab et al. (2018)	Yes	Yes	Yes	Yes	Yes	No	Yes	Yes	Yes	Very valuable
Albutt et al. (2021)	Yes	Yes	Yes	Yes	Yes	Yes	Yes	Yes	Yes	Very valuable
Alshowkan and Gamal (2019)	Yes	Yes	Yes	Yes	Yes	No	Yes	Yes	Yes	Very valuable
Barr et al. (2019)	Yes	Yes	Yes	Yes	Yes	Yes	Yes	Yes	Yes	Very valuable
Berg et al. (2020a, 2020b)	Yes	Yes	Yes	Yes	Yes	Yes	Yes	Yes	Yes	Very valuable
Hagen et al. (2017)	Yes	Yes	Yes	Yes	Yes	No	Yes	Yes	Yes	Very valuable
Hamaideh (2017)	Yes	Yes	Yes	Yes	Yes	No	Yes	Yes	Yes	Very valuable
Hylén et al. (2018)	Yes	Yes	Yes	Yes	Yes	No	Yes	Yes	Yes	Very valuable
Kanerva et al. (2015)	Yes	Yes	Yes	Yes	Yes	No	Yes	Yes	Yes	Very valuable
Maddineshat et al. (2023)	Yes	Yes	Yes	Yes	Yes	Yes	Yes	Yes	Yes	Very valuable
Marshall et al. (2019)	Yes	Yes	Yes	Yes	Yes	No	Yes	Yes	Yes	Very valuable
Olasoji et al. (2024)	Yes	Yes	Yes	Yes	Yes	Yes	Yes	Yes	Yes	Very valuable
Sollied et al. (2023)	Yes	Yes	Yes	Yes	Yes	No	Yes	Yes	Yes	Very valuable
True et al. (2017)	Yes	Yes	Yes	Yes	Yes	No	Yes	Yes	Yes	Very valuable
Vahidi et al. (2018a, 2018b)	Yes	Yes	Yes	Yes	Yes	Yes	Yes	Yes	Yes	Very valuable
Vahidi et al. (2018a, 2018b)	Yes	Yes	Yes	Yes	Yes	Yes	Yes	Yes	Yes	Very valuable
Vogt et al. (2024)	Yes	Yes	Yes	Yes	Yes	Yes	Yes	Yes	Yes	Very valuable

**TABLE 4:** Quality assessment of all selected studies based on Mixed Methods Appraisal Tool (MMAT) (Hong et al. 2018).

	1. Are there clear research questions?	2. Do the collected data allow addressing the research questions?	3. Is the qualitative approach appropriate to answer the research question?	4. Are the qualitative data collection methods adequate to address the research question?	5. Are the findings adequately derived from the data?	6. Is the interpretation of results sufficiently substantiated by data?	7. Is there coherence between qualitative data sources, collection, analysis, and interpretation?
Fallahi‐Khoshknab et al. (2018)	Yes	Yes	Yes	Yes	Yes	Yes	Yes
Albutt et al. (2021)	Yes	Yes	Yes	Yes	Yes	Yes	Yes
Alshowkan and Gamal (2019)	Yes	Yes	Yes	Yes	Yes	Yes	Yes
Barr et al. (2019)	Yes	Yes	Yes	Yes	Yes	Yes	Yes
Berg et al. (2020a, 2020b)	Yes	Yes	Yes	Yes	Yes	Yes	Yes
Hagen et al. (2017)	Yes	Yes	Yes	Yes	Yes	Yes	Yes
Hamaideh (2017)	Yes	Yes	Yes	Yes	Yes	Yes	Yes
Hylén et al. (2018)	Yes	Yes	Yes	Yes	Yes	Yes	Yes
Kanerva et al. (2015)	Yes	Yes	Yes	Yes	Yes	Yes	Yes
Maddineshat et al. (2023)	Yes	Yes	Yes	Yes	Yes	Yes	Yes
Marshall et al. (2019)	Yes	Yes	Yes	Yes	Yes	Yes	Yes
Olasoji et al. (2024)	Yes	Yes	Yes	Yes	Yes	Yes	Yes
Sollied et al. (2023)	Yes	Yes	Yes	Yes	Yes	Yes	Yes
True et al. (2017)	Yes	Yes	Yes	Yes	Yes	Yes	Yes
Vahidi et al. (2018a, 2018b)	Yes	Yes	Yes	Yes	Yes	Yes	Yes
Vahidi et al. (2018a, 2018b)	Yes	Yes	Yes	Yes	Yes	Yes	Yes
Vogt et al. (2024)	Yes	Yes	Yes	Yes	Yes	Yes	Yes

### 2.5 Data Extraction and Synthesis

The results from all the included studies were extracted by copying and pasting them into a Word document. Data synthesis was carried out using thematic analysis (Clarke and Braun 2016). All the results of the included studies were subjected to initial first‐level coding up to the sentence level. Initial coding results were condensed into second‐level descriptive codes, which were subsequently grouped into similar themes. All recurring and common themes that emerged from the included studies were analyzed to identify the major themes. Two reviewers independently conducted the data extraction, coding, and thematic analysis. Discrepancies were resolved by comparing independent coding and through further discussions.

## 3 Results

**FIGURE 2:**
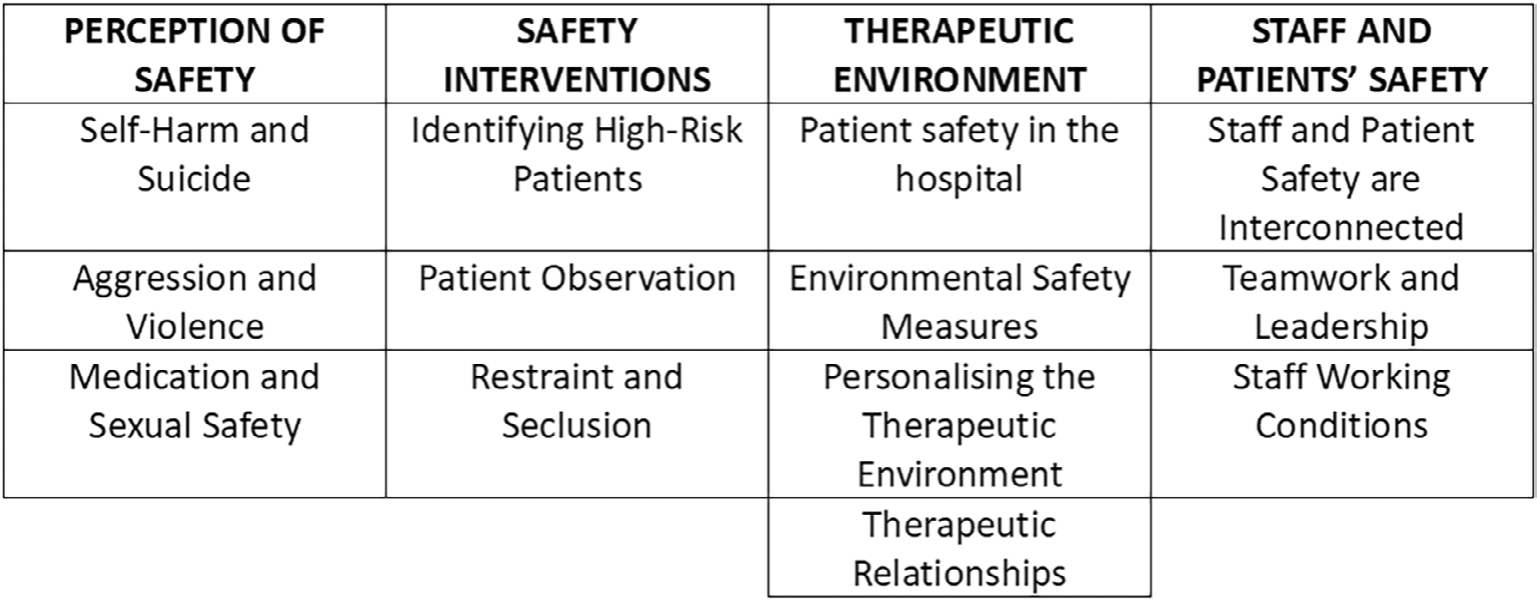
The four major themes and 13 subthemes identified from this review.

The findings are organized into four major themes (Perception of safety, Safety interventions, therapeutic environment, staff and patients' safety) and 13 subthemes (Figure 2) described below.

### 3.1 Perception of Safety

Perception of safety is discussed under three subthemes (self‐harm and suicide, aggression and violence, medication and sexual safety).

#### 3.1.1 Self‐Harm and Suicide

Nurses consider safety around self‐harm to be a vital but challenging aspect of care (Kanerva et al. 2015). Possible self‐harm and suicide attempts are seen as critical factors in maintaining a secure care setting, particularly for patients with suicidal ideation, depression, or a history of self‐harm, who are considered high‐risk and require careful observation (Alshowkan and Gamal 2019; Vahidi et al. 2018a, 2018b).

Suicide is viewed as a possible outcome for various patient groups, making thorough risk assessment extremely important, especially during new admissions (Alshowkan and Gamal 2019; Hagen et al. 2017).

“I could not exclude any psychiatric patient as safe from the risk of suicidal thought. Assessment of newly admitted patients for suicidal risk is an essential procedure for any psychiatric patient.” (Alshowkan and Gamal 2019)

Hospitals implement suicide protocols, including removing potentially harmful items upon admission (Alshowkan and Gamal 2019). Despite tools like checklists and forms, staff note their limitations in capturing patients' mental states and non‐verbal cues (Berg et al. 2020a, 2020b). Ultimately, trust, collaboration, and a calm presence are seen as essential to managing suicide risk effectively (Berg et al. 2020a, 2020b; Hagen et al. 2017).

Nurses' risk assessments tend to be more flexible and holistic, incorporating subtle or unreported indicators (Berg et al. 2020a, 2020b). Nurses also evaluate risk through interactions, behavioral observations, and professional assessments, relying on more than verbal clues while intuition and clinical judgment play a vital role in recognizing subtle signs like unhappiness or isolation (Berg et al. 2020a, 2020b; Hagen et al. 2017; Vahidi et al. 2018a, 2018b).

Psychological safety is influenced by patient distress and is dependent on physical safety (Vogt et al. 2024). Mental health nurses support patient safety by addressing psychological distress, fostering optimism, and guiding patients toward change through therapeutic interactions (Barr et al. 2019). Understanding suicidal thoughts, emphasizing the value of life, and maintaining hope are key to achieving this (Barr et al. 2019; Hagen et al. 2017).

Uncertainty and fear of liability persist, with staff often feeling powerless or guilty after patient suicides (Berg et al. 2020a, 2020b; Hagen et al. 2017). Emotional responses such as sadness and shame are common, but nurses often suppress these feelings to remain composed and maintain patient safety (Berg et al. 2020a, 2020b). Coping mechanisms include emotional detachment, peer support, and occasional breaks (Berg et al. 2020a, 2020b).

#### 3.1.2 Aggression and Violence

Managing patient aggression while preserving a therapeutic environment requires confidence, non‐judgment, and adaptable boundaries (Barr et al. 2019). Nurses identify aggression and violence as the second most significant patient safety risk, after medication errors (Alshowkan and Gamal 2019). A positive ward environment contributes to improved safety (Kanerva et al. 2015).

Patients at risk of self‐harm or aggression require close monitoring, and nurses must intervene to protect patients from harming themselves or others (Alshowkan and Gamal 2019). Nurses check for concealed or hazardous items and may restrict cosmetics or personal belongings (Fallahi‐Khoshknab et al. 2018; Alshowkan and Gamal 2019; Maddineshat et al. 2023; Vahidi et al. 2018a, 2018b).

“One protocol that we follow is that we take a patient's belongings during admission and make sure that he didn't hide any objects that may harm him or others.” (Alshowkan and Gamal 2019)

Aggressive patients pose significant safety and legal risks when adequate protection for staff is lacking, and staff expressed concern about legal consequences, particularly in managing male patients (Maddineshat et al. 2023).

While male staff were expected to manage violence, some believed female caregivers, especially older women, were more effective at de‐escalating aggression through empathy and a non‐threatening tone (Hylén et al. 2018). Male nurses prefer de‐escalation and diversion (Alshowkan and Gamal 2019). Repeated exposure to workplace aggression increases stress and reduces morale (Barr et al. 2019). Verbal abuse and fear of assault heighten anxiety, leading staff to avoid patients and dread shifts (Barr et al. 2019). Physical strain and fear of injury further impair their ability to work (Barr et al. 2019). Staffing shortages hinder care, and disruptive behaviors negatively affect the ward environment (Alshowkan and Gamal 2019; True et al. 2017).

#### 3.1.3 Medication and Sexual Safety

Medications in psychiatric settings pose serious risks, including misuse, overdose, and suicide (Fallahi‐Khoshknab et al. 2018; Alshowkan and Gamal 2019). Nurses enforce safety protocols, monitor ingestion and side effects, and verify proper administration to reduce errors and enhance patient safety (Hagen et al. 2017; Kanerva et al. 2015; Vahidi et al. 2018a, 2018b).

“If a patient is very dangerous in terms of the safety, I will inform my colleagues to be careful when they want to give medications to the patient because he/she may overuse them or use them wrongly.” (Fallahi‐Khoshknab et al. 2018)

Nurses view the care team as responsible for maintaining sexual safety, which is a key aspect of patient care (Olasoji et al. 2024). Shared spaces, lighting, and privacy all influence safety, and although not standard, patients should be informed of their individual expectations (Olasoji et al. 2024). Tags help limit access and support ward security (Olasoji et al. 2024).

“I know, I've gotten reports from consumers that they feel uncomfortable sharing bathrooms. So, there's just some environmental things that make it a bit difficult.” (Olasoji et al. 2024)

### 3.2 Safety Interventions

This theme, safety interventions, is discussed under three subthemes (identifying high‐risk patients, patient observation, restraint, and seclusion).

#### 3.2.1 Identifying High‐Risk Patients

Patient safety involves identifying and caring for high‐risk individuals, particularly those who may pose harm to themselves or others (Fallahi‐Khoshknab et al. 2018; Vahidi et al. 2018a, 2018b). Nurses assess safety levels at admission through patient history, records, and physical assessments to understand needs and detect pre‐existing wounds that may lead to infection or legal issues (Fallahi‐Khoshknab et al. 2018; Maddineshat et al. 2023; Vahidi et al. 2018a, 2018b).

“At the time of hospitalization, the status of the patient is checked in terms of suicide, medication allergy, escape, assault, provocations and aggression, history of medication abuse, and seizure disorder; in general, patients are identified with regard to safety issues.” (Fallahi‐Khoshknab et al. 2018)

Vulnerable patients require additional attention. For example, patients with disabilities are escorted to communal areas to ensure their safety (Fallahi‐Khoshknab et al. 2018). In forensic settings, new or unmedicated patients are seen as potential risks due to unpredictable behaviors (Marshall et al. 2019). Staff emphasized the need to distinguish between criminality and mental illness to provide fair, safe care (Barr et al. 2019). Concerns about patient aggression that is unaddressed highlight risks to staff safety (Marshall et al. 2019).

Emotional intelligence supports safer interactions by helping staff manage responses to patient histories, and clinical supervision improves communication and reduces bias (Barr et al. 2019; Sollied et al. 2023). Staff often distrust self‐reports, relying instead on intuition to detect suicide risk, though this can be fallible (Berg et al. 2020a, 2020b; Hagen et al. 2017). Some staff believe intuitive actions have prevented suicides even when distress wasn't verbalized (Hagen et al. 2017).

Opinions on safety protocols vary; some prefer structured guidelines, while others emphasize the need for adaptability in unpredictable situations (Hylén et al. 2018; Marshall et al. 2019).

#### 3.2.2 Patient Observation

Direct observation is preferred over camera‐based monitoring, with staff viewing it as the most effective method for evaluating patients (Fallahi‐Khoshknab et al. 2018). Even with surveillance cameras, direct observation remains essential, particularly at night, when nurses check on patients every 20–30 min and intervene if needed (Fallahi‐Khoshknab et al. 2018). Continuous observation is key to ensuring patient safety on psychiatric wards, and its absence has been linked to serious incidents, such as suicide due to monitoring failure (Fallahi‐Khoshknab et al. 2018).

Nurses assess patients after handover, focusing on those needing special attention and taking safety steps when unusual events are detected (Vahidi et al. 2018a, 2018b). Their familiarity with patients enables rapid responses and helps predict risks based on patient behavior, ward traffic, and the environment (Vahidi et al. 2018a, 2018b). High‐risk patients are placed near the nursing station for close monitoring (Fallahi‐Khoshknab et al. 2018), and staff maintain vigilance, especially when patients exhibit harmful behaviors such as irritability (Maddineshat et al. 2023).

On the other hand, prioritizing documentation over observation can result in missed signs of risk (Alshowkan and Gamal 2019). Practical observation requires maintaining visibility and restoring dignity while ensuring safety (Berg et al. 2020a, 2020b). Nurses often anticipate changes through subconscious signals, which can reduce incidents of aggression (Sollied et al. 2023).

Staff viewed patient safety as something that demands increased attention, especially at night when risky behaviors increase (Fallahi‐Khoshknab et al. 2018). Nurses monitor sleep patterns and intervene promptly to prevent harm (Fallahi‐Khoshknab et al. 2018; Sollied et al. 2023). Sustained, direct observation is central to preventing harm and ensuring patient safety (Fallahi‐Khoshknab et al. 2018; Alshowkan and Gamal 2019; Berg et al. 2020a, 2020b; Maddineshat et al. 2023; Sollied et al. 2023; Vahidi et al. 2018a, 2018b).

“I am personally sensitive to after twelve o'clock at night and inspect the room with my flashlight. I monitor the sleep pattern of the patients walking along the corridor in order to know why they are still awake.” (Fallahi‐Khoshknab et al. 2018)

#### 3.2.3 Restraint and Seclusion

Shifting from custodial to relational practices improved safety by reducing the need for restraint and seclusion and promoting dignity in patient care (Marshall et al. 2019). Although restraint and seclusion are often used without patient agreement and may involve increased force (Alshowkan and Gamal 2019), staff supported decreasing their use while acknowledging the associated risks (Barr et al. 2019). On the other hand, staff emphasized respectful restraint, monitoring, and acknowledged the risks for both patients and staff (Alshowkan and Gamal 2019; Barr et al. 2019; Hylén et al. 2018). Staff avoided restraint in some cases because it can endanger both the patient and staff applying it (Barr et al. 2019). Despite this, staff in psychiatric inpatient settings maintain that mechanical restraint remains a necessary tool for maintaining safety (Hylén et al. 2018).

“No one wants to restrain, it puts you at risk as soon as you put your hands on the patient.” (Barr et al. 2019)

### 3.3 Therapeutic Environment

Therapeutic environment is discussed under four subthemes (patient safety in the hospital, environmental safety measures, personalizing the therapeutic environment, therapeutic relationships).

#### 3.3.1 Patient Safety in the Hospital

Patient safety in psychiatric settings encompasses both physical protection and psychological security, influenced by environment, staff conduct, and patient conditions (Alshowkan and Gamal 2019; Kanerva et al. 2015). Nurses consider a safe ward environment crucial for patient safety and safe care delivery (Alshowkan and Gamal 2019; Kanerva et al. 2015). Nurses found that ward design and facilities supported patient safety (Alshowkan and Gamal 2019).

A calm, structured environment improves patient participation and reduces distress (Sollied et al. 2023). Similarly, a strong safety culture, embedded in staff training, leadership, and open communication, supports incident prevention and fosters a collective responsibility for care quality (True et al. 2017).

Observing aggression or privacy breaches from peers can heighten patient insecurity (Kanerva et al. 2015; Sollied et al. 2023). Nurses noted that patients closely watch each other and react to environmental changes, indicating heightened sensitivity to safety cues (Sollied et al. 2023).

“Safety is not only about not being afraid of physical threats—when you think about the reasons many patients come here, there is a kind of fear caused by the patient's own thoughts.” (Kanerva et al. 2015)

In psychiatric wards, nurses view patient vulnerability, medication side effects, and the potential for harm, such as self‐harm, violence, or escape, as reasons for heightened vigilance (Vahidi et al. 2018a, 2018b). Nurses must accurately interpret subtle signs, particularly when patients lack insight or refuse treatment (Vahidi et al. 2018a, 2018b). Experience, presence, and environmental knowledge underpin safe practice (Vahidi et al. 2018a, 2018b). Mental health staff must assess risk, personalize protective measures, and balance safety with autonomy, as both overuse and underuse of constraints can harm patients, especially during crises (Berg et al. 2020a, 2020b; Maddineshat et al. 2023).

Rising severity in mental and physical health conditions among patients compounds safety risks, particularly when combined with substance abuse (Albutt et al. 2021). Also, safety concerns vary across settings: inpatient care focuses on physical harm, community care emphasizes relapse triggers and medication adherence (Albutt et al. 2021).

#### 3.3.2 Environmental Safety Measures

Creating a secure environment is crucial for vulnerable and trauma‐affected patients, yet such areas are not consistently available in hospital wards (Olasoji et al. 2024). The ward design should allow safe staff movement and observation (Kanerva et al. 2015) as poor ward design, including unsafe nursing stations, increases safety concerns (Alshowkan and Gamal 2019; Kanerva et al. 2015; Maddineshat et al. 2023).

“The ward design doesn't permit the nurses to carefully observe the patients. The rooms should more be larger to allow patients and staff to move easily.” (Alshowkan and Gamal 2019)

Staff emphasized the importance of secure windows, doors, and seclusion rooms (Alshowkan and Gamal 2019). Escape attempts are common due to involuntary admissions and lack of illness awareness (Alshowkan and Gamal 2019). Patients exploit environmental lapses, such as accessing hazardous items or hiding small objects (Vahidi et al. 2018a, 2018b).

Physical space limitations lead to overcrowding, noise, and aggression (Maddineshat et al. 2023). Nurses stress the importance of outdoor areas, privacy, and entertainment to reduce fatigue and agitation (Kanerva et al. 2015; Maddineshat et al. 2023).

Equipment misuse poses risks, as patients may use it for self‐harm, even under supervision (Vahidi et al. 2018a, 2018b). Equipment modifications, such as safety glass, have been shown to reduce aggression‐related injuries (Maddineshat et al. 2023; Marshall et al. 2019). Infection control and resource availability directly affect safe care (Alshowkan and Gamal 2019; Kanerva et al. 2015).

Smoking policies were introduced to manage air quality and mitigate conflict, but the Covid‐19 pandemic disrupted care, reduced activities, and increased safety risks due to patient non‐compliance and limitations in respiratory care (Maddineshat et al. 2023). Fire safety is a concern as false alarms can lead to escapes (Alshowkan and Gamal 2019). Staff advocate for fire‐resistant materials and safe flooring to prevent accidents (Kanerva et al. 2015).

#### 3.3.3 Personalizing the Therapeutic Environment

Empathy, compassion, and hope are essential for personalized care, and nurses are encouraged to recognize each patient's strengths and work collaboratively to ensure optimal outcomes (Barr et al. 2019). Individualized clinical pathways balance risk and vigilance, with varied approaches depending on the healthcare professional's background (Berg et al. 2020a, 2020b). Recognizing that suicidal patients require diverse, tailored strategies, doctors focused on treatment customization, while psychologists highlighted understanding personal experiences and emotional regulation to support safety (Berg et al. 2020a, 2020b).

“I work with the individual patients' underlying feelings about suicidality… Through gaining insight, the patients find other ways to express their emotions.” (Berg et al. 2020a, 2020b)

Therapeutic environments were adjusted to meet individual needs, with structured routines calming high‐risk behaviors and flexibility ensuring responsiveness to change (Berg et al. 2020a, 2020b). Personalized safety plans included early warning signs, coping strategies, and support systems; however, they were sometimes delayed or implemented generically, limiting effectiveness (Berg et al. 2020a, 2020b).

Understanding patients' histories, triggers, and coping strategies fostered psychological safety and allowed for personalized care (Marshall et al. 2019; Vogt et al. 2024). Staff relied on knowledge of individual behaviors, constant observation, and calm settings to anticipate and mitigate risk (Sollied et al. 2023). Recognizing behavioral shifts enabled early intervention and de‐escalation (Sollied et al. 2023; Vahidi et al. 2018a, 2018b).

Safety involved collaboration between staff and patients. Nurses and patients jointly evaluated suicide risk, with decisions shaped by direct interaction and multidisciplinary input (Berg et al. 2020a, 2020b). Yet, staff faced tension between risk documentation and meaningful communication (Berg et al. 2020a, 2020b). Healthcare professionals maintained safety through therapeutic connections, privacy, and responsiveness to patient preferences, ranging from verbal reassurance to quiet presence (Berg et al. 2020a, 2020b; Kanerva et al. 2015).

#### 3.3.4 Therapeutic Relationships

Patient safety is closely tied to the quality of patient‐staff relationships (Hylén et al. 2018; Vogt et al. 2024). These relationships, built on respect, honesty, and open‐mindedness, help staff recognize emotional instability and de‐escalate risks (Barr et al. 2019; Kanerva et al. 2015; Sollied et al. 2023). Positive, friendly interactions allow staff to monitor health and address behavioral issues, especially in patients with trauma or self‐destructive behaviors (Hylén et al. 2018; Sollied et al. 2023).

“Building that therapeutic relationship is important; treating people with respect, being honest and immediate; being open‐minded and respectful; tolerant and not fiery.” (Barr et al. 2019)

Staff presence and meaningful engagement, through games, meals, or conversations, were seen as crucial to reducing violence and ensuring safety (Hylén et al. 2018; True et al. 2017). Therapeutic engagement, especially during therapy groups, created connections and safety for both patients and staff (Vogt et al. 2024). Kindness, trust, and remaining calm during interactions helped prevent violent incidents and supported recovery (Hylén et al. 2018; Sollied et al. 2023). Communication was viewed as a preventive and responsive tool during and after violent behavior, helping patients reflect and reduce recurrence (Hylén et al. 2018).

In high‐security or trauma‐related care, staff employed patience, trust, and respect to maintain safety, helping patients process distressing experiences (Sollied et al. 2023). Trust was essential in suicide risk assessments, where professionals gradually explored suicidal thoughts through relationship‐building (Berg et al. 2020a, 2020b). Staff also highlighted the importance of listening to patient concerns, including risks, medication errors, or safety threats (Vahidi et al. 2018a, 2018b).

Permanent staff responsibilities, brief psychiatrist visits, and a lack of patient contact from psychologists were identified as barriers to safe care (Maddineshat et al. 2023; Vogt et al. 2024). A stable, communicative team was considered vital for maintaining safety (Vogt et al. 2024).

### 3.4 Staff and Patients' Safety

Staff and patients' safety is discussed under three subthemes (staff and patient safety is interconnected, teamwork and leadership, staff working conditions).

#### 3.4.1 Staff and Patient Safety Are Interconnected

Nurse and patient safety are closely linked (Alshowkan and Gamal 2019; Hamaideh 2017). Nurses believe their safety is essential for working efficiently and that patients' safety involves protection from self‐harm, harm from other patients, and potential risks posed by staff (Kanerva et al. 2015). Interacting with psychiatric patients can jeopardize staff safety, causing stress, fatigue, and time off work, especially when patients are uncooperative or admitted involuntarily (Alshowkan and Gamal 2019). Nurses often feel unsafe, viewing distress and risk as part of the job (Vogt et al. 2024). Staff describe trauma from ward work, constant fear of adverse events, and experiences of verbal and physical abuse (Vogt et al. 2024). Trauma can impair decision‐making and lead to the use of restraint instead of de‐escalation (Vogt et al. 2024). Supporting staff is vital, as coping with trauma alone is harmful (Vogt et al. 2024). Alarm systems were seen as essential to staff safety and mental well‐being (Kanerva et al. 2015).

“We didn't really have the time to make patients feel safe… we didn't even have a minute spare.” (Vogt et al. 2024)

#### 3.4.2 Teamwork and Leadership

Patient safety culture is closely tied to teamwork, staffing, continuous improvement, and management support (Hamaideh 2017). Effective teamwork and leadership are essential for providing safe nursing care, built on trust, effective communication, and collaboration (Barr et al. 2019; Marshall et al. 2019).

Supportive teams enabled staff to express uncertainty, share risks, and maintain professional integrity under stress (Berg et al. 2020a, 2020b; Sollied et al. 2023). Structured supervision and team support increased psychological safety (Vogt et al. 2024). When teamwork, leadership, and communication were effective, staff could deliver safer, more compassionate care (Kanerva et al. 2015; Marshall et al. 2019; Sollied et al. 2023).

Communication, especially during handovers, and staff collaboration were key to managing patient aggression and emotional distress (Maddineshat et al. 2023; Sollied et al. 2023). Debriefing after critical incidents supports patient safety by reducing staff stress, addressing emotional responses, and enhancing risk management, team morale, and care quality (Sollied et al. 2023). However, when emotions are overlooked or sessions skipped, debriefs lose effectiveness, impacting staff well‐being and patient care (Sollied et al. 2023; Vogt et al. 2024).

System‐level issues, including staffing shortages, bureaucracy, and inadequate resources, were identified as significant threats to safety (True et al. 2017). Inadequate staffing, especially reliance on temporary workers, raised safety concerns due to limited integration, experience, and trust (Vogt et al. 2024). Leadership inconsistencies and poor team composition also affected patient care and safety (Barr et al. 2019).

Most nurses acknowledged the head nurse's crucial role in ensuring patient safety, serving as a model for care, and maintaining a safe environment for staff and patients (Alshowkan and Gamal 2019). Head nurses support staff by promoting patient collaboration, therapeutic relationships, and safety planning based on individual needs (Alshowkan and Gamal 2019). They guide new staff, share safety protocols, and emphasize the ward's safety culture during orientation (Alshowkan and Gamal 2019). Their skill in managing patients with cognitive impairments and maintaining composure reassures staff in challenging situations (Alshowkan and Gamal 2019).

“Our role model in dealing with and treating patients is our head nurse, as she is very caring about establishing and maintaining ward, staff and patient safety.” (Alshowkan and Gamal 2019)

Leadership backing and team reviews are crucial for patient safety, as staff value mutual and managerial support, especially following violent events (Berg et al. 2020a, 2020b; Hylén et al. 2018). However, nurses often report lacking formal support, and essential mental health skills are overlooked due to insufficient leadership (Berg et al. 2020a, 2020b; True et al. 2017). During crises, management's responses often lack empathy or practical aid, instead focusing on fault‐finding (Vogt et al. 2024). Despite support for anonymous reporting, a blame culture persists, discouraging concerns due to fear of job repercussions (True et al. 2017; Vogt et al. 2024).

Senior management often lacked understanding of mental health care needs and failed to promote psychological safety (Vogt et al. 2024). Management's poor understanding is correlated with failures to prioritize physical safety, as malfunctioning alarms and broken fixtures jeopardize safety and erode staff morale (Vogt et al. 2024). Management's limited practical ward knowledge impedes understanding of staff trauma and operational challenges (Vogt et al. 2024).

A division exists between ward staff and senior management, with the latter often unavailable and dismissive, fostering an “us versus them” mentality that undermines psychological safety (Vogt et al. 2024). Safety complaints from mental health nurses frequently go unaddressed (Maddineshat et al. 2023). Staff call for a culture that promotes psychological safety, where leaders accept all feedback without defensiveness, encourage learning from incidents, and demonstrate greater managerial appreciation (Vogt et al. 2024).

#### 3.4.3 Staff Working Conditions

Staffing deficits lead to feelings of lack of physical and psychological safety, ineffective de‐escalation methods, and increased patient aggression, thereby endangering both patients and staff (Vogt et al. 2024). Understaffing also damages staff‐patient relationships and prevents patients from feeling safe (Marshall et al. 2019; Vogt et al. 2024). Frontline staff frequently felt overwhelmed by workload, pressured to complete tasks, and worked unpaid overtime, all of which posed safety concerns (Albutt et al. 2021). Inadequate staffing, heavy workloads, and excessive documentation hinder the delivery of quality psychiatric care and individualized attention (Alshowkan and Gamal 2019; Maddineshat et al. 2023).

“Say I guess some of the struggles with staffing if we are given say that one extra staff at that staff at that time it is very beneficial it's like giving a little bit of a sigh of relief we have that one extra person it‐one person makes a big difference.” (Marshall et al. 2019)

Nurses in forensic mental health expressed enthusiasm for working with complex patients in challenging but engaging environments requiring constant risk assessments to ensure safety (Barr et al. 2019). However, nurses' performance is often evaluated based on administrative tasks rather than quality of care, leading to the prioritization of metrics over patient safety (Albutt et al. 2021). Colleague and managerial support are critical in this demanding environment, where staff often feel vulnerable and overextended (Hylén et al. 2018). Lack of motivation and limited time prevent nurses from performing thorough mental status exams and daily patient interviews (Maddineshat et al. 2023).

Staff communication is crucial and can significantly influence safety by preventing escalation, but poor communication can escalate violence (Marshall et al. 2019; Hylén et al. 2018). Information governance rules can restrict access to patient information communication within and outside mental health services, which can negatively impact patient safety (Albutt et al. 2021). However, there is a need to protect client confidentiality by limiting access to sensitive data only to those who require it (Albutt et al. 2021).

Experience, expertise, and unit knowledge increase staff's perceived safety and competence (Marshall et al. 2019; Hylén et al. 2018). Specialist knowledge of offending behaviors in individuals with acute mental illness and proactive problem‐solving skills is essential (Barr et al. 2019). Nurses report a need for better training in managing patient aggression, suggesting the implementation of regular workshops (Alshowkan and Gamal 2019). Ongoing education, even for experienced staff, refreshes strategies to prevent violence and improve patient handling (Hylén et al. 2018). Untrained staff handling emergencies or restraints may worsen situations (Hylén et al. 2018). Effective violence management requires regular training, support, and rapid response to restore safety (Sollied et al. 2023).

“We are not trained in the ward, but we depend in our academic study—I think that if a training workshop or courses were conducted periodically for nurses, it would help us.” (Alshowkan and Gamal 2019)

## 4 Discussion

This review has highlighted four major themes that present staff's perspective on patient safety in psychiatric wards. These major themes are: (1) Perception of safety around self‐harm, suicide, aggression, violence, medication, and sexual issues for example, Alshowkan and Gamal (2019), Berg et al. (2020a, 2020b), (2) safety interventions around identifying, observing, and restraining patients for example, Fallahi‐Khoshknab et al. (2018), Vahidi et al. (2018a, 2018b), (3) therapeutic environment around the hospital, safety measures, personalisation, and relationships for example, Alshowkan and Gamal (2019), Kanerva et al. (2015), and (4) staff and patient safety are related for example, Alshowkan and Gamal (2019), Vogt et al. (2024). This review is a follow‐up to a previous study that explored patients' perspectives about safety on psychiatric wards (Akinlotan et al. 2025).

This review has demonstrated that nurses perceive safety as a complex and emotionally challenging issue, involving constant vigilance, teamwork, and intuition to balance physical, psychological, and sexual safety for example, Berg et al. (2020a, 2020b), Olasoji et al. (2024). Safety has been described by staff as emotionally complex in a previous study (Kirk 2024). Nurses reported that, while managing patient safety is the primary goal, maintaining a balance between physical and psychological safety is a challenging task (Cui et al. 2021). For example, while restraints enhance safety, they also negatively impact patient comfort, creating a distressing trade‐off (Cui et al. 2021). Similarly, sexual activity may be voluntary, but can still pose a safety risk, as many psychiatric inpatients are cognitively impaired and unable to give proper consent (Cuomo et al. 2021). Furthermore, nurses felt that they played a critical role in managing self‐harm, aggression, and medication misuse on psychiatric wards through restricting access to harmful objects and constant observation (Fallahi‐Khoshknab et al. 2018; Barr et al. 2019). Previous studies have explored medication and substance abuse from a patient's perspective, which described witnessing other patients stockpiling medications for misuse and abuse (Rahman et al. 2022; Strike et al. 2020). Staff perceive physical safety on mental health wards to be threatened by patient aggression and environmental defects such as poor lighting and cramped spaces (Haines et al. 2018), whilst psychological safety is undermined when physical risks are perceived, and sexual safety is jeopardized by unwanted comments or assaults (Jackson 2020).

This review has found that staff interventions involve continuous, individualized assessment and direct observation of high‐risk patients to prevent harm, managing aggression, and responding to emerging risks (Fallahi‐Khoshknab et al. 2018; Vahidi et al. 2018a, 2018b). A previous study has highlighted the benefits of constant observation in preventing self‐harm or harming others (Reen et al. 2020). Other studies have recognized that continuous observation significantly invades patients' privacy (Anstee et al. 2024; Barnicot et al. 2017). Bowers and Park (2002) and Saigle and Racine (2018) state that whilst safety must take priority in acute risk situations, efforts to minimize privacy intrusion can help maintain dignity without undermining protective measures. Also, the presence of suicidal behavior on admission was reported to be the most critical predictor of emerging risks in inpatient settings (Karasch et al. 2020), and therefore, staff's ability to identify high‐risk patients on admission could reduce the risk of patients dying from suicide (Prestmo et al. 2023).

According to this review, a therapeutic environment in psychiatric care integrates safety measures with personalized and compassionate interactions (Berg et al. 2020a, 2020b; Sollied et al. 2023). Berg et al. (2020a, 2020b) noted that tailoring treatment to each patient's specific needs and circumstances is essential for providing safe clinical practice for patients with complex mental health problems (Berg et al. 2020a, 2020b). Moreover, all staff agreed that an open psychiatric ward setting can reduce conflict between patients and improve communication and support (Efkemann et al. 2019). On the other hand, this review emphasized the need for a secure ward, featuring locked windows, controlled access doors, and designated seclusion spaces, to prevent patients from leaving the ward (Alshowkan and Gamal 2019). The review also highlighted that recovery is supported through intentional environmental design, vigilant monitoring, and shared approaches to risk management (Alshowkan and Gamal 2019). For example, nurses have noted that rooms should be more spacious to facilitate safer and easier movement for both patients and staff, thereby enhancing observation and reducing risk (Alshowkan and Gamal 2019). This is supported by Lavender et al. (2015), who noted that limited ward space poses a challenge for staff, leading to reduced job performance and increased physical effort.

This review has shown that the safety of staff and patients in mental health settings is interconnected (Alshowkan and Gamal 2019; Kanerva et al. 2015). Another study also found this to be true, indicating that patients perceive staff and patient well‐being as interdependent (Lin et al. 2025). When staff are stressed or disengaged, patients often feel neglected or humiliated, which can lead to conflict and isolation (Sattar et al. 2024; Akinlotan and Jalo 2024). Respectful, supportive interactions are essential for the safety and well‐being of both staff and patients (Lu et al. 2022). Furthermore, this review specifically highlighted that protecting staff well‐being through proper support, training, and leadership directly influences their ability to maintain patient safety and deliver effective care (Marshall et al. 2019; Sollied et al. 2023; True et al. 2017).

Bruno and Bracco (2016) reported that when healthcare professionals work under conditions that support their mental, emotional, and physical well‐being, they are better able to maintain high standards of patient safety. Another study has found a direct link between low psychological safety and increased job demands, leading to negative impacts on work performance (Amoadu et al. 2025).

The summary of the findings from this review indicates that patient safety on psychiatric wards is multifaceted, necessitating a balance between protection and autonomy, effective environmental design, compassionate care, and staff well‐being. Safety interventions must consider both patients' needs and the emotional and physical demands on staff to create a therapeutic and secure environment (Price et al. 2018).

## 5 Clinical Implications and Recommendations

Patient safety on psychiatric wards is a multifaceted challenge requiring targeted strategies to prevent self‐harm, suicide, violence, aggression, sexual abuse, and medication misuse (e.g., Kirk 2024). Clinically, these findings highlight the necessity of early identification of high‐risk patients, especially those exhibiting suicidal behavior upon admission, as this is a possible predictor of future incidents (e.g., Fallahi‐Khoshknab et al. 2018). To ensure safety, continuous individualized assessments and direct observation must be standard practice, enabling early intervention and risk prevention (e.g., Sollied et al. 2023). Environmental changes that reduce access to harmful objects and collaborative risk management create safer therapeutic spaces, and this should be considered (e.g., Barr et al. 2019). Also, patients' treatment plans should be personalized to address each patient's unique needs, support recovery, and reduce risk. Protecting staff well‐being through comprehensive training, supportive leadership, and mental health resources is also critical (Akinlotan 2024), as staff resilience directly impacts the quality of patient care (e.g., Berg et al. 2020a, 2020b). To enhance patient safety, psychiatric units must implement structured protocols for ongoing risk assessment, invest in staff development, and encourage a culture of open communication (e.g., Olasoji et al. 2024; Akinlotan 2024). These measures collectively strengthen the capacity to manage complex risks, promoting safer and more effective psychiatric care environments.

## 6 Strengths and Limitations

This is the first systematic review that examines safety on psychiatric wards from the perspective of the healthcare professionals. A notable strength of this systematic review is its synthesis of current knowledge on patient safety in psychiatric wards, offering an up‐to‐date and comprehensive overview. The use of the PRISMA protocol ensures methodological rigor, while an exhaustive search across six databases reduces the risk of missing relevant studies. Additionally, two quality assessment tools (CASP and MMAT) were used, enhancing reliability. The studies included in this review come from several countries across different continents and cultures, which provides a more global perspective on patient safety, accounting for variations in healthcare systems and practices. Moreover, this review explores a diverse range of healthcare professionals, which enriches the understanding of patient safety by incorporating multiple viewpoints and roles within psychiatric care. However, a significant limitation is the exclusion of studies published in non‐English languages. This could potentially overlook crucial international research and affect the generalizability of the conclusions in non‐English speaking countries.

## 7 Conclusion

This study has provided a reliable summary of the current evidence on staff's perspectives regarding safety on psychiatric wards. Based on a comprehensive search of six electronic databases, 17 studies that met the eligibility criteria were subjected to thematic analysis. Four major themes were identified as influencing staff perspectives on patient safety in psychiatric wards: perception of safety, safety interventions, therapeutic environment, and staff and patient safety. Nurses perceive safety as a complex and emotionally challenging responsibility. Staff interventions require continuous, individualized assessment and observation. Therapeutic environments integrate safety with compassionate care. Staff and patient safety are closely interconnected. The summary of the findings indicates that patient safety on psychiatric wards is multifaceted, necessitating a balance between protection and autonomy, effective environmental design, compassionate care, and staff well‐being.

## 8 Relevance to Clinical Practice

Clinically, findings from this review highlight the necessity of early identification of high‐risk patients, especially those exhibiting suicidal behavior upon admission (e.g., Fallahi‐Khoshknab et al. 2018), implementation of environmental changes that reduce access to harmful objects, and collaborative risk management to create safer therapeutic spaces for both patients and staff (e.g., Barr et al. 2019). These measures will collectively strengthen the capacity to manage complex risks and promote safer and more effective psychiatric care environments.

## Author Contributions

**Oladapo Akinlotan:** conceptualization, methodology, funding acquisition, project administration, writing – review and editing, supervision. **Maria Dumitriu:** formal analysis, investigation, data curation, writing – draft

## Funding

This work was supported by Anglia Ruskin University.

## Ethics Statement

The authors have nothing to report.

## Conflicts of Interest

The authors declare no conflicts of interest.

## Supporting information


**Appendix S1:** PRISMA checklist.

## Data Availability

The data that support the findings of this study are available on request from the corresponding author. The data are not publicly available due to privacy or ethical restrictions.

## Appendix A

### Phase 1: Assessing Relevance

State your overview/guideline question (target question) and the question being addressed in the review being assessed:

**Table:**

Category	Target question (e.g., overview or guideline)	Review being assessed
Patients/Population(s):	Patients in psychiatric settings	Patients in psychiatric settings
Exposure(s) and comparator(s):	Patient safety	Patient safety
Outcome(s):	Staff perspectives	Staff perspectives
Does the question addressed by the review match the target question?	Yes	

### Phase 2: Identifying Concerns with the Review Process

**Table:**

Domain 1: Study Eligibility Criteria	
Describe the study eligibility criteria, any restrictions on eligibility and whether there was evidence that objectives and eligibility criteria were pre‐specified:	
1.1 Did the review adhere to pre‐defined objectives and eligibility criteria?	Yes
1.2 Were the eligibility criteria appropriate for the review question?	Yes
1.3 Were eligibility criteria unambiguous?	Yes
1.4 Were any restrictions in eligibility criteria based on study characteristics appropriate (e.g., date, sample size, study quality, outcomes measured)?	Yes
1.5 Were any restrictions in eligibility criteria based on sources of information appropriate (e.g., publication status or format, language, availability of data)?	Yes
Concerns regarding specification of study eligibility criteria	Low
Rationale for concern	No

Domain 2: Identification and Selection of Studies	
Describe methods of study identification and selection (e.g., number of reviewers involved):	
2.1 Did the search include an appropriate range of databases/electronic sources for published and unpublished reports?	Yes
2.2 Were methods additional to database searching used to identify relevant reports?	Yes
2.3 Were the terms and structure of the search strategy likely to retrieve as many eligible studies as possible?	Yes
2.4 Were restrictions based on date, publication format, or language appropriate?	Yes
2.5 Were efforts made to minimize error in selection of studies?	Yes
Concerns regarding methods used to identify and/or select studies	Low
Rationale for concern	No

Domain 3: Data Collection and Study Appraisal	
Describe methods of data collection, what data were extracted from studies or collected through other means, how risk of bias was assessed (e.g., number of reviewers involved) and the tool used to assess risk of bias:	
3.1 Were efforts made to minimize error in data collection?	Yes
3.2 Were sufficient study characteristics available for both review authors and readers to be able to interpret the results?	Yes
3.3 Were all relevant study results collected for use in the synthesis?	Yes
3.4 Was risk of bias (or methodological quality) formally assessed using appropriate criteria?	Yes
3.5 Were efforts made to minimize error in risk of bias assessment?	Yes
Concerns regarding methods used to collect data and appraise studies	Low
Rationale for concern	No

Domain 4: Synthesis and Findings	
Describe synthesis methods:	
4.1 Did the synthesis include all studies that it should?	Yes
4.2 Were all pre‐defined analyses reported or departures explained?	Yes
4.3 Was the synthesis appropriate given the nature and similarity in the research questions, study designs and outcomes across included studies?	Yes
4.4 Was between‐study variation (heterogeneity) minimal or addressed in the synthesis?	Yes
4.5 Were the findings robust, for example, as demonstrated through funnel plot or sensitivity analyses?	Yes
4.6 Were biases in primary studies minimal or addressed in the synthesis?	Yes
Concerns regarding the synthesis and findings	Low
Rationale for concern	No

### Phase 3: Judging Risk of Bias

Summarize the concerns identified during the Phase 2 assessment:

**Table:**

Domain	Concern	Rationale for concern
1. Concerns regarding specification of study eligibility criteria	Low	No
2. Concerns regarding methods used to identify and/or select studies	Low	No
3. Concerns regarding methods used to collect data and appraise studies	Low	No
4. Concerns regarding the synthesis and findings	Low	No

Risk of Bias in the Review	
Describe whether conclusions were supported by the evidence:	
A. Did the interpretation of findings address all of the concerns identified in Domains 1 to 4?	Yes
B. Was the relevance of identified studies to the review's research question appropriately considered?	Yes
C. Did the reviewers avoid emphasizing results on the basis of their statistical significance?	Yes
Risk of bias in the review risk	Low
Rationale for risk	No

Risk of bias analyses for the review (ROBIS) tool (Whiting et al. 2016).
